# Was Hahnemann Inspired?

**Published:** 1888-02

**Authors:** M. W. Van Denburg

**Affiliations:** Fort Edward, N. Y.


### WAS HAHNEMANN INSPIRED?

One who reads the recent articles pro and con in the current homœopathic journals must often be led to ask himself this question: Does any difference of opinion arise? What says the *Organon?* To the law and the testimony, this is an end to all dispute! "The frequently recurring expression of different opinions, how best to practice Homœopathy, can easily be settled by referring to the teachings of Hahnemann." Can they, indeed? On what ground, gentlemen, please tell us? Was Hahnemann inspired? Or did he write, think, observe, and labor like other men, like, for example, you, my brother? If he did, then how is it that he has become infallible? What is your line of reasoning? This much praised *Organon* has many good things in it, and many very foolish ones. From Section 9 to Section 20, inclusive, is one string of undemonstrable hypotheses. Section 25 is pure gold. The less said about Hahnemann's mesmerism the better. However, our point may be noted in Section 2 of this part of the *Organon.* This says the master, "this (mesmeric pass) is a great adjuvant to homœopathic medicines in the actual treatment of the entire disease."

It seems, then, that Hahnemann himself was not above the use of adjuvants. Shall we read HIM out of the fraternity?

Now, it may all be true, that "the more ignorant the physician the oftener will it be his duty to give Opium or Morphia, and, if not ignorant, the duty to give it will *never* arise." Will any one point out a physician who is not ignorant? Where does the omniscient doctor reside? Are there many such in this country? Unfortunately, the large bulk of the rich have to depend on physicians who are human and limited in their capacity. The physician is called not always solely to cure the disease. Often the patient asks, first of all, to be relieved in the shortest possible time from his suffering; he is willing to be treated later for the remoter effects. Now, imagine a man rolling and howling with pain from a renal calculus, and being told if he will wait patiently for an hour or two in this agony, he may expect the doctor will find the right simillimum if it exists in the materia medica, and then in the course of another hour or two, he may hope for relief, when the administration of the proper amount of Morphia will, inside of ten minutes, begin to ease the brain. If the patient knows this fact, he will not be long in selecting the proper doctor. It is impossible for me to believe that the "old veterans cured all such cases without adjuvant treatment," for the simple reason that I think they occasionally found one that was incurable. Old veterans and far-off times have a great charm for many, because the faults and failures have dropped out and only a history of the good remains. Most of them were men of like passions with ourselves, and, other things being equal, just as fallible.

In this discussion, I wish to avoid dogmatic assertions as far as possible. I am not so confident, because I do not know *all* about it, that an incipient case of gonorrhœa is "an erroneously supposed local disease." Until more is known in what this disease consists, I prefer to reserve my decision, and hence will not argue that point.

We often upbraid the folly of those of "our friends, the enemy," who cannot see that a cure is ever effected by a homœopathic physician. Who ascribe it all to his medicative nature or coincidences? Who will never try a homœopathic remedy in a homœopathic way because it is Homœopathy? Who would rather let a patient suffer on and die, even, than to treat them homœopathically? There are those whose prejudices carry them to such an extent, and they are to be both censured and pitied. It seems very like such a prejudice as this that some of our homœopathic brethren have for Opium or Morphia.

M. W. VAN DENBURG, A. M., M. D.

FORT EDWARD, N. Y.

